# Mechanically-Induced Solid-State Reaction for Fabrication of Soft Magnetic (Co_75_Ti_25_)_100−x_B_x_ (x: 2, 5, 10, 15, 20, 25 at%) Metallic Glassy Nanopowders

**DOI:** 10.3390/molecules25153338

**Published:** 2020-07-23

**Authors:** Mohamed Sherif El-Eskandarany, Naser Ali

**Affiliations:** Nanotechnology and Applications Program, Energy and Building Research Center, Kuwait Institute for Scientific Research, Safat 13109, Kuwait; nmali@kisr.edu.kw

**Keywords:** mechanical alloying, high-energy ball milling, solid-state reaction, glass-transition, crystallizations, super-cooled liquid region, thermodynamics

## Abstract

Metallic glasses, with their short-range order structure, exhibit unique characteristics that do not exist in the corresponding crystalline alloys with the same compositions. These unusual properties are attributed to the absence of translational periodicity, grain boundaries, and compositional homogeneity. Cobalt (Co)-based metallic glassy alloys have been receiving great attention due to their superior mechanical and magnetic properties. Unluckily, Co-Ti alloys and its based alloys are difficult to be prepared in glassy form, due to their rather poor glass-forming ability. In the present work, the mechanical alloying approach was employed to investigate the possibility of preparing homogeneous (Co_75_Ti_25_)_100−x_B_x_ starting from elemental powders. The feedstock materials with the desired compositions were high-energy ball-milled under argon atmosphere for 50 h. The end products of the powders obtained after milling revealed a short-range order structure with a broad amorphization range (2 at% ≤ B ≤ 25 at%). The behaviors of these glassy systems, characterized by the supercooled liquid region, and reduced glass transition temperature, were improved upon increasing B molar fraction. The results had shown that when B content increased, the saturation magnetization was increased, where coercivity was decreased.

## 1. Introduction

Life in the 21st century cannot depend on limited groups of materials; instead, it is dependent on unlimited families of advanced materials [1]. Despite the traditional categories of materials, which may not completely match with the modern industrial requirements, a rather newcomer so-called “metallic glass” [2] has found an important space in the functional classifications of metals and metal alloys [3]. Metallic glasses, with their short-range order structure, possess exciting properties which are of interest not only for basic solid-state physics, but also for metallurgy, surface chemistry, and technology [4,5]. These properties are quite different from crystalline materials (long-range order) metals, making them promising candidates for technical applications. The high mechanical ductility and yield strength, unusual corrosion resistance, high magnetic permeability, and low coercive forces are some of those desirable properties found in metallic glassy alloys [6,7,8,9]. 

There are different approaches used for preparing amorphous and metallic glassy alloys, such as the atomic disordering of crystalline lattices, solid-state amorphization reaction between pure elements, and solid-state transformations from metastable phase [10]; however, they are exclusively synthesized by rapid solidification (RS) of melts or vapors, using the melt spinning technique [2]. In the melt spinning process, the melt of metal alloy is rapidly cooled with a very high cooling rate, reaching 10^6^ K/s [10]. Unluckily, this simple process has some limitations and cannot be employed for preparations of metallic glasses in immiscible systems or for those systems that have positive values in their enthalpy change of formation (∆H^for^). Moreover, RS cannot be used with those systems that have a drastic gap difference between their melting points, such as Al (661 °C) and Ta (3020 °C) [11].

Apart from RS approach, a wide range of amorphous and metallic glassy alloys have been prepared by mechanical alloying (MA) technique that was established by Koch et al. in 1983, when they prepared amorphous Ni_60_Nb_40_ powders by milling a mixture of pure elements [12]. In contrast to the melt spinning technique, MA is a typical solid-state process, in which the reaction takes place between the diffusion couples of the powders (reactant material) at ambient temperature (being very closed to room temperature) [13]. Since then, the MA process has been employed for preparing those systems that are difficult or impossible to be obtained by the RS and the melting and casting techniques [1].

Within the last decade, Co-based metallic glassy alloys have received great attention due to their very useful soft magnetic properties. Besides, they have stimulated much interesting research based on their ultrahigh strength, particularly bulk metallic glass (BMG). This is attributed to their excellent soft magnetic behaviors [6,7,9,14,15,16,17]. Ferromagnetic Co-metallic glassy alloys possess very small magnetocrystalline anisotropy, as well as (near) zero magnetostriction [8], which is responsible for their exceptional soft magnetic behaviors. This is indexed by their very low coercivity, extremely low core loss, and a moderate saturation magnetization [18]. Based on these excellent characteristics, Co-based metallic glasses have become the best choices for several electromagnetic industrial applications, such as pulse compression applications, flexible antenna, keyless entry systems, power and current transformers, spike blockers, magnetic shields, and sensors [8]. Moreover, Co-based metallic glassy alloys offer the opportunity to decrease transformer core losses [7]. In particular, a large elastic flexibility guarantees excellent insensitivity concerning plastic deformations and a small electrical conductivity reduces the eddy-current losses [17]. Unfortunately, due to their poor glass-forming ability (GFA) and the absence of any deep eutectic compositions in the equilibrium phase diagram, it is very difficult to prepare Co-based metallic glassy alloys [18].

The present study was carried out in part to fabricate novel metallic glassy-(Co_75_Ti_25_)_100−x_B_x_ (x: 0–25 at%) nanopowders via MA technique, using high-energy ball milling (BM). In this work, the GFA of this new ternary system has been investigated as a function of boron (B) additives, in the range between 2 at% and 30 at%. Furthermore, the effect of B-concentration on the thermal stability and magnetic properties of the Co-Ti base is reported. The detailed chemical analysis, conducted by inductively coupled plasma (ICP) spectrometry of the starting composition is given in at% in Table 1. The iron contamination analyzed by the ICP technique was in the range between 0.05 to 0.23 at%.

## 2. Results and Discussion

### 2.1. Morphology and Crystal Structure 

**Figure 1:**
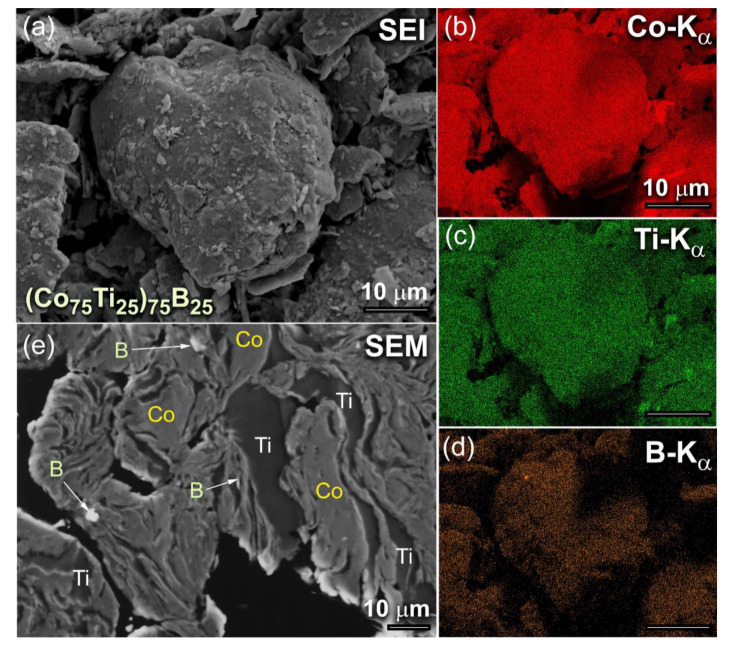
(**a**) Scanning electron image (SEI) of as-BM (Co_75_Ti_25_)_75_B_25_ powders obtained after 6 h of MA time. The corresponding EDS-elemental maps of Co, Ti, and B are displayed in (**b**), (**c**) and (**d**), respectively. The FE-SEM micrograph of the cross-sectional view for the powders after this stage of milling is presented in (**e**).

The typical morphological characteristics of (Co_75_Ti_25_)_100−x_B_x_, exemplified by (Co_75_Ti_25_)_75_B_25_ powders, obtained after 6 h of MA time, is presented in Figure 1. The powder particles, trapped between colliding grinding balls were experienced from excessive welding during the BM process, where metallic Co and Ti were severely plastically deformed and agglomerated to form larger powders of more than 100 μm in diameter, as presented in Figure 1a. These aggregated particles composited of Co, Ti, and B, as confirmed by the energy-dispersive X-ray spectroscopy (EDS)-elemental mapping, as shown in Figure 1b–d, respectively. At this early stage of mechanical alloying (MA), the composition of the milled powders significantly varied from particle to particle and within the individual particles themselves. The field-emission scanning electron microscope (FE-SEM) micrograph of the cross-sectional view for the powders presented in Figure 1a is displayed in Figure 1e. The powders revealed lamellar-like metallography, composited of thick Co, Ti metallic layers, where the fine B particles were dispersed into these layers, as indexed in Figure 1e.

**Figure 2:**
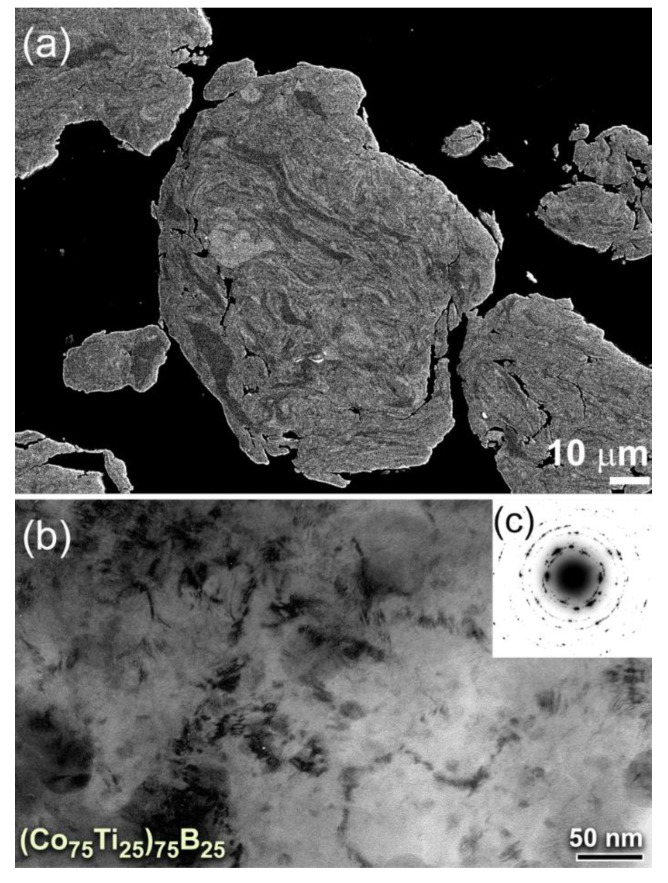
(**a**) FE-SEM micrograph of the cross-sectional view for (Co_75_Ti_25_)_75_B_25_ powders obtained after 18 h of MA time. The corresponding bright-field image (BFI) and selected area diffraction pattern (SADP) of the powders after this stage of MA are displayed in (**b**) and (**c**), respectively.

Figure 2a displays the FE-SEM micrograph of the cross-sectional view for the powder particles obtained after 18 h of MA time. The metallic Co/Ti lamella became thinner, indicating a continuous improvement in the internal homogeneity of composition, as shown in Figure 2a. Formation of these thin-layers accelerated the solid-state diffusion that occurred at their fresh interfaces to form a more homogeneous composition. Meanwhile, the powder particles were continuously subjected to severe lattice imperfections, indexed by plastic deformation, and lattice and point defected, as shown in Figure 2b. After this intermediate stage of MA, the powders consisted of polycrystalline grains of the starting materials with no evidence for the formation metallic glassy phase, as characterized by those sharp spots that appeared in the selected area diffraction pattern (SADP) (Figure 2c).

**Figure 3:**
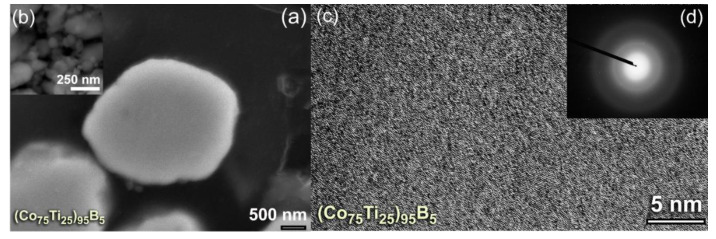
(**a**) High-magnification FE-SEM micrographs of (**b**) the cross-sectional view and (**b**) morphology for (Co_75_Ti_25_)_75_B_25_ powders obtained after 50 h of MA time. The FE-HRTEM and nanobeam diffraction pattern (NBDP) of the powders after this stage of MA are displayed in (**c**) and (**d**), respectively.

With further MA time (50 h), the powders were disintegrated into finer particles (Figure 3a,b), where the internal structure of the powders continues to be refined. After 50 h of MA time, the lamella-like metallography completely disappeared, as shown in the high-magnification FE-SEM presented in Figure 3a. This implies the completion of the MA process and the formation of a single homogeneous phase. The powders of this final product possessed desirable morphological characteristics of being ultrafine (ranged from 120 nm to 250 nm in diameter) and had spherical-like morphology with smooth surfaces, as displayed in Figure 3b. The field emission high-resolution transmission electron microscope (FE-HRTEM) image of the final product obtained after 50 h of MA time (Figure 3c) revealed featureless maze-like morphology of an amorphous phase. Besides, the corresponding nanobeam diffraction pattern (NBDP) showed a typical amorphous-like halo-diffuse pattern, as displayed in Figure 3d.

**Figure 4:**
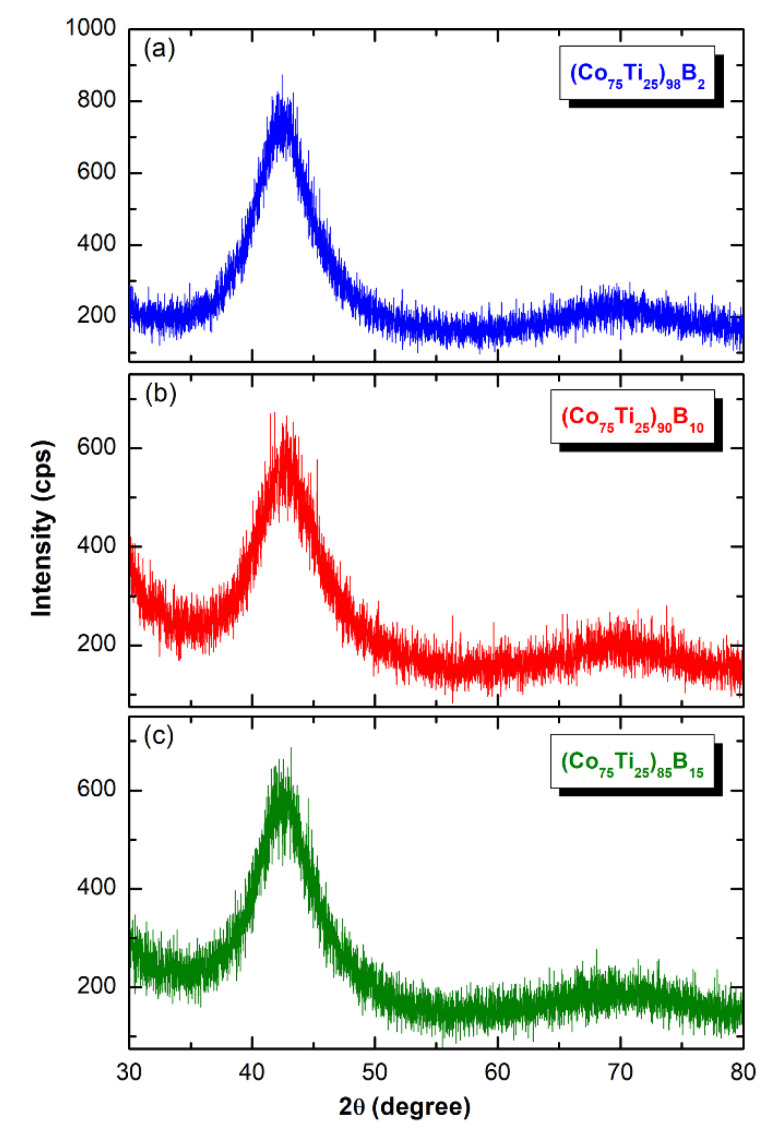
X-ray diffraction (XRD) patterns of MA (**a**) (Co_75_Ti_25_)_98_B_2_, (**b**) (Co_75_Ti_25_)_90_B_10_, and (**c**) (Co_75_Ti_25_)_85_B_15_ powders, obtained after 50 h of BM.

The X-ray diffraction (XRD) patterns of MA-(Co_75_Ti_25_)_100−x_B_x_ (x: 2, 10, and 15 at%) obtained after 50 h of BM time are displayed collectively in Figure 4. All the patterns exhibited only broad diffuse haloes, in scattering range of 2θ between 40–50°, with no indication of any unprocessed crystalline phases. This implying the capability of MA approach to fabricate the desired amorphous alloy powders in a wide amorphous range.

**Figure 5:**
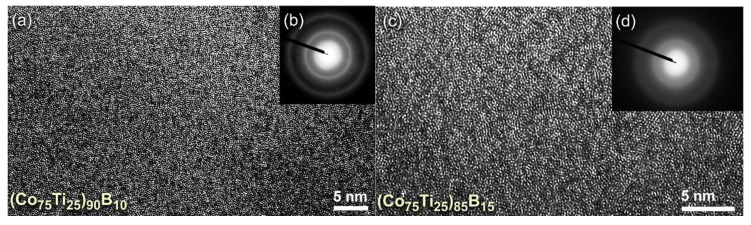
(**a**) FE-HRTEM, and (**b**) NBDP of (Co_75_Ti_25_)_90_B_10_ powders obtained after 50 h of MA time (end-product). The FE-HRTEM and corresponding NBDP for the end-product of (Co_75_Ti_25_)_85_B_15_ are displayed together in (**c**) and (**d**), respectively.

The FE-HRTEM micrographs taken for the end-products of (Co_75_Ti_25_)_90_B_10_, and (Co_75_Ti_25_)_85_B_15_ are displayed together with their NBDPs in Figure 5a,b and Figure 5c,d, respectively. The powders had typical fine morphology with random atomic distribution (Figure 5a,c), indicating the formation of short-range order structure. Furthermore, the NBDPs displayed spot-free halo-diffuse pattern, suggesting the absence of long-range order (crystalline phases) and medium-range order (metastable phase), as presented in Figure 5b,d.

**Figure 6:**
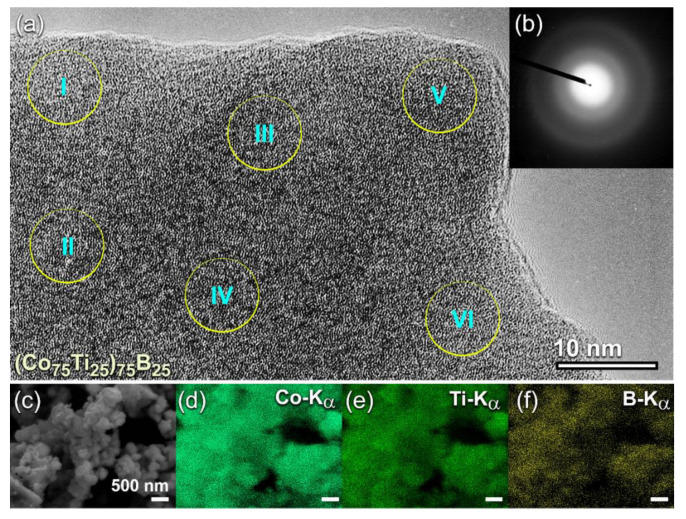
(**a**) FE-HRTEM, and (**b**) NBDP of (Co_75_Ti_25_)_75_B_25_ powders obtained after 50 h of MA time. The corresponding FE-SEM micrograph is shown in (**c**) together with the elemental EDS-mapping of (**d**) Co, (**e**) Ti, and (**f**) B. The circular symbols indexed with a Roman number (I, II, III, IV, V, and VI) in (**a**) refer to the zones selected for conducting local (~5nm in diameter) EDS elemental analysis. The analytical results are listed in Table 2.

To understand the degree of homogeneity in the chemical composition for the end-products (50 h), intensive FE-HRTEM/EDS investigations were conducted for all the samples. The FE-HRTEM micrograph of (Co_75_Ti_25_)_75_B_25_ powders, which implies the formation of amorphous structure (Figure 6a) with the absence of the crystalline phase (Figure 6b), was classified into six local zones (~5 nm for each) to investigate the local composition beyond the atomic level. The results of the EDS analysis, which is presented in Table 2 have indicated a high degree of homogeneity in the elemental composition and the absence of compositional degradation. The average chemical composition of this sample was very close to the real composition of the starting Co_56.28_Ti_18.79_B_24.93_ powders (Table 1). The as-processed powders obtained after this end-point of MA (50 h) consisted of ultrafine spherical particles with sizes ranged between 200 nm to 500 nm in diameter, as shown in Figure 6c).

**Figure 7:**
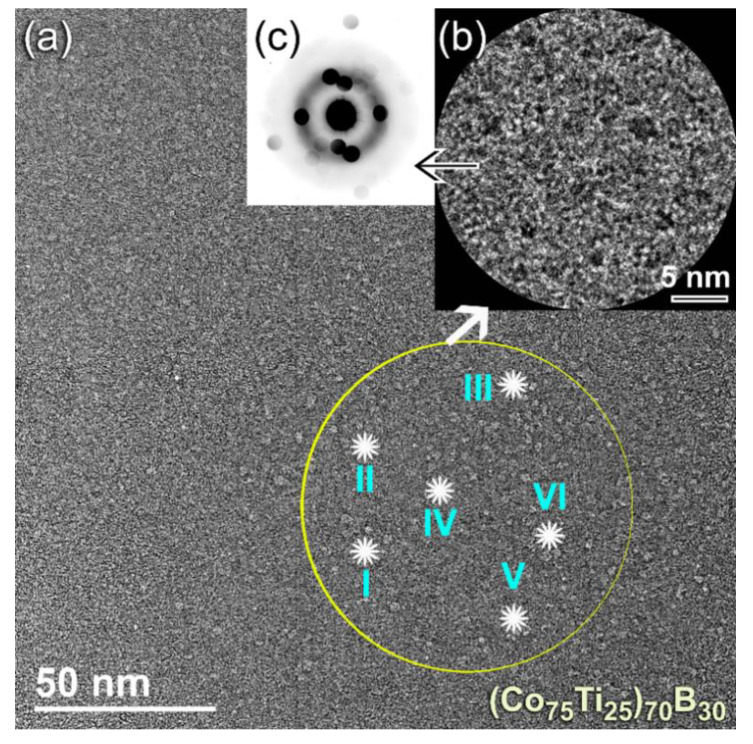
(**a**) FE-HRTEM of (Co_75_Ti_25_)_70_B_30_ powders obtained after 50 h of MA time, (**b**) atomic resolution transmission electron microscope (TEM) image taken from the zone indexed by a circular symbol in (**a**), and (**c**) NBDP corresponding to (**b**). The results of EDS-elemental analysis for the spots indexed by Roman numbers (I, II, III, IV, V, and VI) are presented in Table 3.

The FE-SEM-EDS elemental maps for the alloying elements of Co (Figure 6d), Ti (Figure 6e), and B (Figure 6f) have indicated that the powers, were homogeneous in the composition without any pieces of evidence of chemical segregations. To realize the glass-forming range of the present ternary system, a further MA experiment with higher B content was carried out. The FE-HRTEM micrograph of (Co_75_Ti_25_)_70_B_30_ powders obtained after 50 h of MA time is presented in Figure 7a. The morphological characteristics of the powders obtained after this stage of milling show a clear contrast in structure when compared with the previous HRTEM images for those samples contained a low concentration of B (≤25 at%). The higher magnification FE-HRTEM image taken for the circular zone indexed in Figure 7a indicated the existence of grey-nano-lenses (~2 to 3 nm in diameter), embedded in the fine amorphous matrix (Figure 7b). The NBDP (Figure 7c) related to the image presented in Figure 7b revealed a halo-diffuse pattern corresponding to the amorphous matrix, overlapped with sharp spots related to unprocessed nanocrystalline B, as shown in Figure 7c. The results indicated the formation of heterogeneous powders, which fluctuated in composition from spot to sport, as presented in Table 3.

### 2.2. Thermal Stability

**Figure 8:**
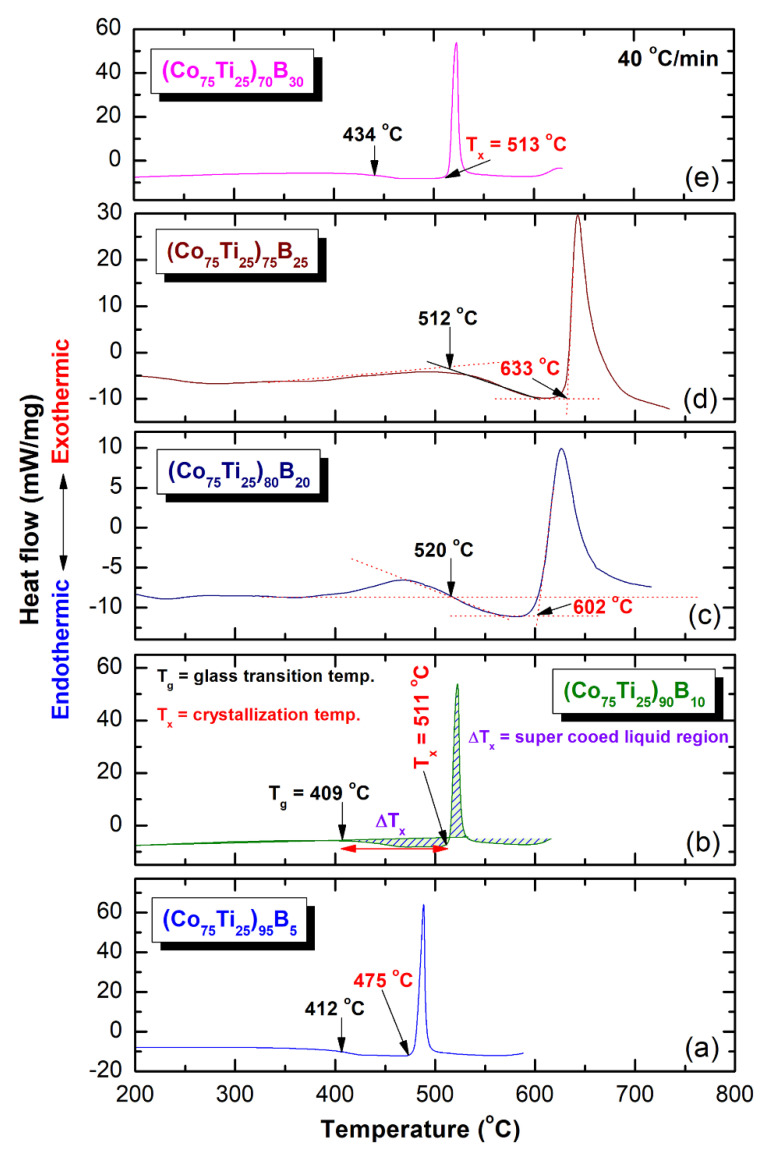
Differential scanning calorimetry (DSC) thermograms of (**a**), (Co_75_Ti_25_)_95_B_5_ (**b**) (Co_75_Ti_25_)_90_B_10_, (**c**) (Co_75_Ti_25_)_80_B_20_, (**d**) (Co_75_Ti_25_)_75_B_25_, and (**e**) (Co_75_Ti_25_)_70_B_30_ powders obtained after 50 of MA time. The corresponding differential thermal analysis (DTA) curves for these systems are presented in Figure 10.

Differential scanning calorimetry (DSC) technique was used to characterize the crystallization behavior of as-MA (Co_75_Ti_25_)_100−x_B_x_ amorphous alloys, indexed by their glass transition temperature (T_g_), crystallization temperature (T_x_), and supercooled liquid region (∆Tx = Tx − Tg). However, differential thermal analysis (DTA) technique was employed to investigate their corresponding melting behaviors, characterized by the melting temperature (T_m_), liquids temperature (T_l_), and reduced glass transition temperature (T_rg_ = T_g_/T_l_). Figure 8a–e present the DSC curves of selected MA-(Co_75_Ti_25_)_100−x_B_x_ powders, while their corresponding DTA traces are displayed in Figure 10a–e. The heating rates used for conducting DSC experiments were 40 °C/min, where it was 10 °C/min for all of DTA experiments. In the DSC measurements, all the samples were isothermally heated up to the desired temperatures before cooling down to room temperature. Then, second heating runs were carried out with the same heating rates to establish baselines.

The DSC thermograms presented in Figure 8a–e exhibited two opposite thermal events, taking place at different temperatures. The onset temperatures for the first events were endothermic, appearing at a low-temperature side in the range between 412 °C to 520 °C, as presented in Figure 8. These endothermic events are related to the T_g_, which is a unique feature of metallic glassy alloys. At this temperature, the solid-amorphous, which is extended from room temperature to T_g_, transformed into liquid-phase (glassy phase) without any structural or compositional changes. The second events, however, were characterized by sharp pronounced exothermic peaks, taken place at a higher temperature (Figure 8), known as crystallization temperature (T_x_). At these T_x_, the metallic glassy phase is crystallized into the long-range order phase. The thermodynamics parameters of T_g_ and T_x_ are used to describe the thermal stability of metallic glassy materials, where ∆T_x_ is used to characterize their GFA. Wide ∆Tx indicates that the system has a good GFA.

**Figure 9:**
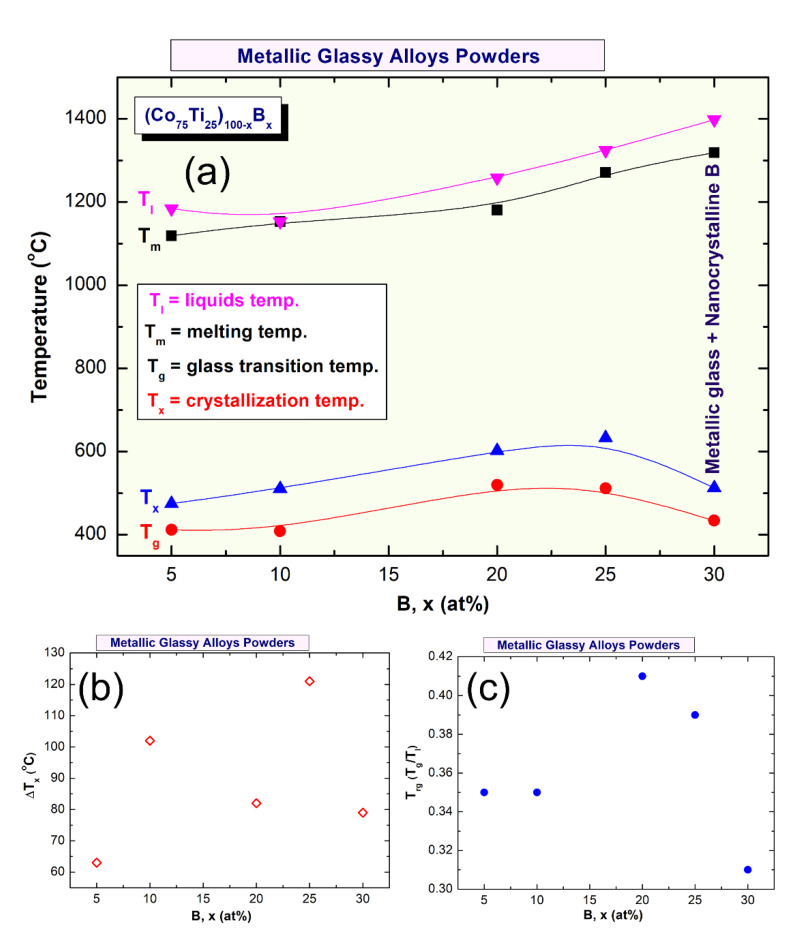
(**a**) Dependence of T_g_, T_x_, T_m_, and T_l_ on B molar fraction of (Co_75_Ti_25_)_100−x_B_x_ metallic glass system. The correlation between ∆T_x_ and T_rg_ and B content are shown in (**b**) and (**c**), respectively.

Depending on B concentrations, metallic glassy (Co_75_Ti_25_)_100−x_B_x_ powders revealed different GFA and crystallization behaviors, as presented in Figure 8 and Figure 9. For instance, (Co_75_Ti_25_)_95_B_5_ glassy powders possessed low values of ∆T_x_ (63 °C) and T_g_ (412 °C), as presented in Figure 8a. This indicates a low GFA and lowers thermal stability of this system when compared with (Co_75_Ti25)_90_B_10_, revealed wider T_x_ (102 °C), and higher T_x_ value (511 °C), as shown in Figure 8b or Figure 9a,b. Increasing the B content (20 at% to 25 at%) led to a monotonical increase in T_x_ (Figure 9a), to be 602 °C and 633 °C, respectively (Figure 8c,d). It should be clarified that ∆T_x_ of (Co_75_Ti_25_)_70_B_20_ has a lower value of (82 °C) when compared with that measured value of (Co_75_Ti_25_)_75_B_25_ (121 °C), as indexed in Figure 8a or Figure 9b.

Based on this comparison, we claim that (Co_75_Ti_25_)_75_B_25_ metallic glassy system revealed the best GFA and possessed high thermal stability. In contrast to these full-metallic glassy systems, nanocomposite (Co_75_Ti_25_)_70_B_30_ powders, which consisted of a glassy phase coexisted with nano-spheres of unprocessed crystalline B particles (Figure 7) revealed lower ∆T_x_ (79 °C) and T_x_ (513 °C) values, as indexed in Figure 8a or Figure 9a,b. The degradation in these thermodynamics parameters may be related to the heterogeneity seen in the chemical composition of (Co_75_Ti_25_)_70_B_30_, as detected by HRTEM/EDS (Figure 7b, Table 3).

**Figure 10:**
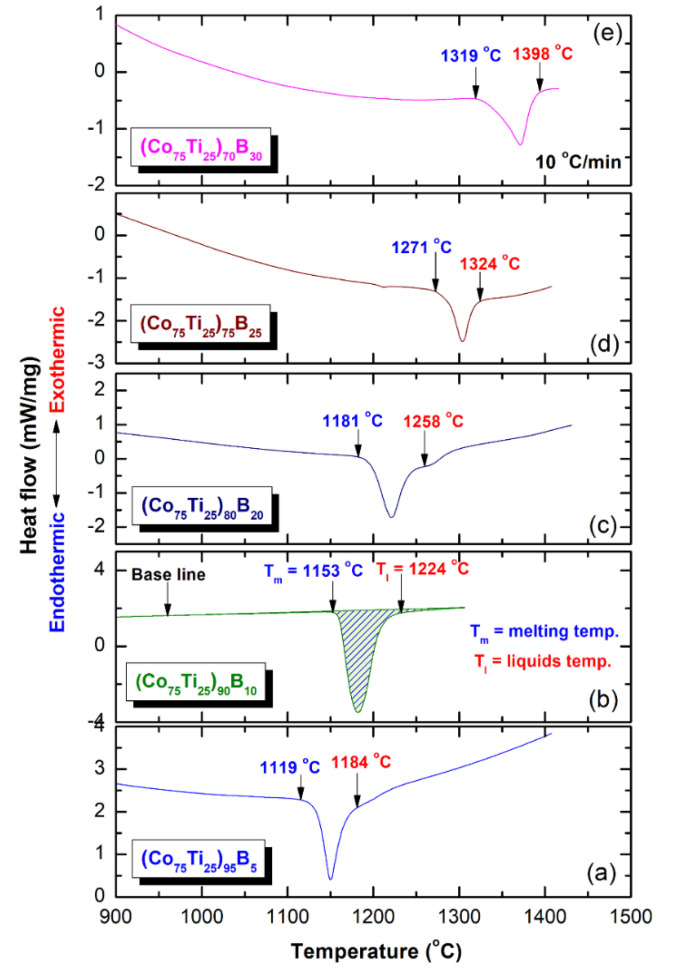
Traces of (**a**), (Co_75_Ti_25_)_95_B_5_ (**b**) (Co_75_Ti_25_)_90_B_10_, (**c**) (Co_75_Ti_25_)_80_B_20_, (**d**) (Co_75_Ti_25_)_75_B_25_, and (**e**) (Co_75_Ti_25_)_70_B_30_ powders obtained after 50 of MA time.

In parallel to ∆T_x_ parameter, the GFA of any metallic glassy alloys produced by rapid solidification technique is described by measuring the melting (T_m_) and liquids (T_l_) temperature, and calculate the T_rg_ = T_g_/T_l_. All the DTA curves of (Co_75_Ti_25_)_100−x_B_x_ systems presented in Figure 10a–e display a single endothermic event for each composition. This endothermic peak revealed an obvious head and tail points, which related to T_m_ and T_l_ temperature, respectively (Figure 10). The onset temperature of T_m_ and T_l_ values measured for the (Co_75_Ti_25_)_95_B_5_ system were 1119 °C and 1184 °C (Figure 9a or Figure 10a), respectively, where the corresponding T_rg_ of this composition was calculated and found to be 0.35 (Figure 9c). However, increasing the B concentration to 10 at% (Figure 10b) led to a significant increase in both T_m_ (1153 °C) and T_l_ (1224 °C), but corresponding T_rg_ of this composition has not been improved and remained at the level of (0.35), as presented in Figure 9c. Both of T_m_ and T_l_ tended to increase with increasing the B content to be 1181 °C and 1268 °C ((Co_75_Ti_25_)_80_B_20_), and 1271 °C and 1324 °C ((Co_75_Ti_25_)_75_B_25_), as presented in Figure 10c,d, respectively. Notably, no improvement in T_rg_ value could be detected, as shown in Figure 9c. The DTA trace of (Co_75_Ti_25_)_70_B_30_ system, which consisted of a glassy phase coexisted with nano-spheres of unprocessed crystalline B particles showed the highest value of T_m_ (1319 °C) and T_l_ (1398 °C), as indexed in Figure 10e. However, this system revealed the lowest value of T_rg_ (0.31), as presented in Figure 9c.

For excellent GFA systems prepared by rapid solidification technique, T_rg_ should be far above 0.5. The current (Co_75_Ti_25_)_100−x_B_x_ system in all range of B content showed low T_rg_ values, laid in the range between 0.31 to 0.41, as displayed in Figure 9c. As a result, the present metallic glassy system is a challengeable system that cannot be easily prepared by the conventional rapid solidification approach.

### 2.3. Magnetic Properties

**Figure 11:**
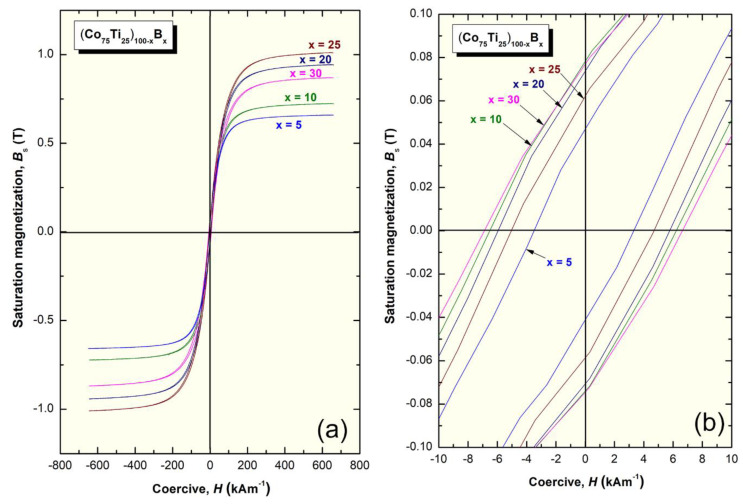
(**a**) Hysteresis B-H loops of (Co_75_Ti_25_)_100−x_B_x_ metallic glassy systems. The Hysteresis was presented with a different scale in (**b**).

**Figure 12:**
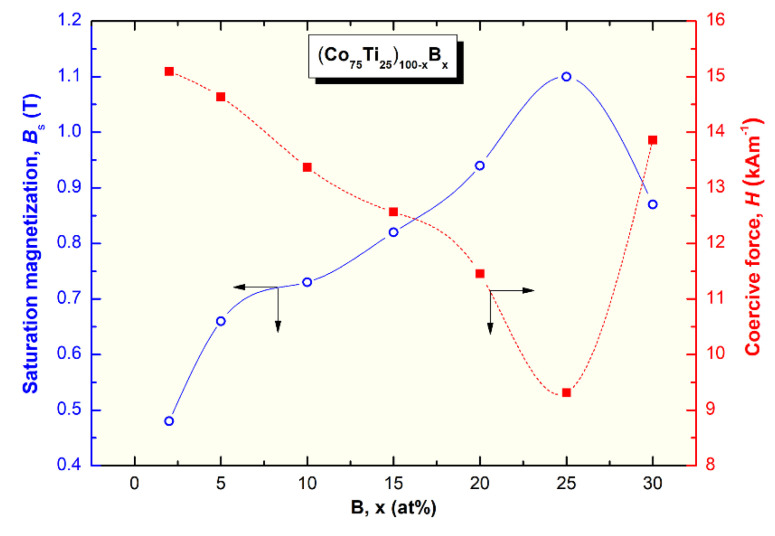
Effect of B content (x, at%) on the saturation magnetization and coercive force of (Co_75_Ti_25_)_100−x_B_x_ metallic glassy systems.

The saturation magnetization (B_s_) and coercive force (H) of (Co_75_Ti_25_)_100−x_B_x_ metallic glassy systems were obtained according to the measured hysteresis loops, as depicted in Figure 11a,b. The loops of all samples exhibited typical soft magnetic behaviors, as can be realized from Figure 11a. The dependence of Bs and H on the B (x content) is presented in Figure 12. Notably, the (Co_75_Ti_25_)_100−x_B_x_ metallic glassy system revealed a rather modest value of B_s_ (0.48 T), with a low coercivity value of about 15 kA m^−1^, as presented in Figure 11b or Figure 12. When B content increased to 5 at%, Bs was increased to 0.66 T, where the H value approached a lower value (14.6 kA m^−1^), indicating an improvement in the soft magnetic characteristics. Further improvement in the increase in Bs was attained upon increasing the concentration B alloying element to 10 at% (0.73 T), 15 at% (0.82 T), and 20 at% (0.94 T), as depicted in Figure 12.

In parallel, the coercivity force, indexed by H was monotonically decreased with increasing the B concentration to be (11.5 kA m^−1^) for (Co_75_Ti_25_)_80_B_20_ (Figure 12). Metallic glassy (Co_75_Ti_25_)_75_B_25_ system possessed the best soft magnetic characteristics, as indexed by its high B_s_ value (1.1 T) and a very low H (9.3 kA m^−1^), as presented in Figure 12. In contrast, the nanocomposite (Co_75_Ti_25_)_70_B_30_ system showed a lower B_s_ value (0.87 T) and a relatively high H value (13.8 kA m^−1^), as indexed in Figure 12. The degradation shown here in the soft magnetic properties of this system was attributed to the heterogeneity in the chemical composition, where unprocessed B nanocrystalline particles have existed (Figure 7, Table 3).

In addition to the current system of this study, there are a considerable number of recent reports which show the possibility of preparing metallic glassy soft magnetic materials through mechanical alloying and other approaches. For example, Co_80−x_Ta_x_Si_5_C_15_ (x = 0, 5) glassy/nanocrystalline system possesses promising soft magnetic behavior, i.e., a minimum coercivity (H_c_) of 1.2 kA m^−1^, which is notably lower than a minimum value obtained for Co_80_Si_5_C_15_ (3.3 kA m^−1^) [19]. Recently, Matsui and Omura reported that Ni-Fe-P alloy exhibited a saturation magnetic flux density of 1.1 T and a coercivity of 8.4 A/m. [20]. They pointed out the impact of good coercivity on the grain refinement by the P alloying, which can result in a lower coercivity [20].

Furthermore, the effect of MA on the magnetic properties of mechanically alloyed Ni_70_Co_30_ has been reported by N. Loudjani et al. [21]. Their results have shown that both the saturation magnetization and coercivity decreased with milling time, attaining the values of 87 emu/g and 30 Oe, respectively, after 25 h of milling [21]. More recently, Zhao et al., have reported the formation of a novel (Fe_0.25_Co_0.25_Ni_0.25_Cr_0.125_Mn_0.125_)_100–x_B_x_ system with B concentration ranged from 9 at% to 13 at%, using melt spinning approach [22]. They pointed out that the system exhibits good soft-magnetic properties: H_c_ = 2.5–7.0 A m^−1^, μ_i_ = 4910–15,830, and B_s_ = 0.40–0.48 T [22]. An interesting study of preparing bulk nano Fe-Si-B-Cu-Nb based alloys by mechanical alloying has been recently reported [23]. This alloy was consolidated into bulk nanocrystalline objects, using spark plasma sintering (SPS) technique. Based on the milling time and composition, the M_s_ and H_c_ were varied from 159.6 to 166.8 emu/g, and 87 to 109.2 Oe, respectively [23].

## 3. Materials and Methods 

### 3.1. Feedstock Materials

High purity elemental powders of Co (150 μm, >99.9 wt%, #266647: Sigma-Aldrich, St. Louis, MO, USA), Ti (45 μm, >99.99 wt%, #366994: Sigma-Aldrich), and B (45 μm, >98 wt%, #GF47837065: Sigma-Aldrich) were used as the starting feedstock materials. The powders were handled, balanced, and then mixed inside the He-atmosphere (99.99%) glove box (UNILAB Pro Glove Box Workstation, mBRAUN, Garching, Germany) to get an amount of 25 g with the desired nominal composition of (Co_75_Ti_25_)_100−x_B_x_ (x: 2–30 at%).

### 3.2. Preparations of Metallic Glassy Alloy Powders

The mixed powders of each composition were individually charged into tool steel vials (500 mL in volume) and well-sealed together and with 75 tool steel balls (11 mm in diameter) in the glove box under He-atmosphere (99.99%), using a ball-to-powder weight ratio was 20:1. The vials were then fixed on a high-energy ball mill (Planetary Mill PULVERISETTE 5, Fritsch, Kitzingen, Germany), where the ball milling (BM) process was carried out for 15, 30, 45, and 60 h at ambient temperature. After each milling run, a small amount (<500 mg) was discharged from the vial for different analyses. Then, the BM process was resumed under the same operating conditions.

### 3.3. Sample Characterizations

The crystal structures of all samples were investigated by X-ray diffraction (XRD) with CuKα radiation, using 9kW Intelligent X-ray diffraction system, provided by SmartLab-Rigaku (Tokyo, Japan). The local structure of the synthesized materials was studied by 200 kV-field emission high-resolution transmission electron microscopy/scanning transmission electron microscopy (HRTEM/STEM) supplied by JEOL-2100F (Tokyo, Japan), and equipped with Energy-dispersive X-ray spectroscopy (EDS) supplied by Oxford Instruments (Abingdon, UK).

The morphological characteristics of the milled and consolidated samples were investigated through field-emission scanning electron microscope (FE-SEM), using 15 kV- JSM-7800F, JEOL (Tokyo, Japan). The local elemental analysis was investigated by energy-dispersive X-ray spectroscopy (EDS, Oxford Instruments, Abingdon, UK) system interfaced with the FE-SEM.

Differential scanning calorimeter (DSC) and differential thermal analysis (DTA), provided by Setaram-France(Caluire, France), using a heating rate of 40 °C/min and 10 °C/min, respectively, employed to investigate the glass transition temperature, glass-forming ability, and thermal stability indexed by the supercooled liquid region and crystallization temperature of the metallic glassy samples.

The magnetization (B_s_) of the as-consolidated samples was measured at room temperature, using a vibrating sample magnetometer (VSM) with a maximum applied magnetic field of 670 kA m^−1^. The coercive force was measured with a B-H loop tracer.

## 4. Conclusions

Due to the absence of deep eutectic compositions in the Co-Ti binary phase diagram, it is very difficult to obtain a metallic glassy phase for this system and its based alloys, using the melting and casting and melt spinning techniques. This is in contrast to the Co-Zr binary system, which possesses several deep eutectic compositions, allowing a wide glass-formation range. The present work has been addressed in part to employ the MA approach for preparing metallic glasses of ternary (Co_75_Ti_25_)_100−x_B_x_ systems in a very wide range of (2 ≤ x ≤ 30 at%), using high-energy ball milling technique. Based on the results of the present work, we can conclude that:

(1) The GFA of (Co_75_Ti_25_)_100−x_B_x_ was improved upon increasing the B molar fraction in the range between 2 at% to 25 at%.
(2) The effect of elemental B of enhancing the GFA is attributed to the chemical bonding between the alloying elements (Co, Ti and B) in the alloy. This can be realized upon considering that electronegativity, which is a very important factor for glass formation, is directly related to the chemical bonding. Electrons of metalloid B element, which transferred to metallic Co-Ti may lead to the formation of very strong covalent bonding and hence led to improve the GFA of (Co_75_Ti_25_)_100−x_B_x_ systems.
(3) Among the as-prepared metallic glassy alloys of (Co_75_Ti_25_)_100−x_B_x_, (Co_75_Ti_25_)_75_B_25_ system revealed excellent thermodynamics properties, as indexed by its high GFA as characterized by the widest ∆T_x_ (121 °C), and high thermal stability, indicated by its highest T_x_ value (633 °C). Moreover, this glassy system revealed the highest T_rg_ value (0.41), which indicates its high GFA when compared with other compositions in the (Co_75_Ti_25_)_100−x_B_x_ systems.
(4) The results have shown that when B content increased, the saturation magnetization was increased to reach to 1.1 T for (Co_75_Ti_25_)_75_B_25_ system, where coercivity was decreased to a very low level of 9.3 kA m^−1^.

## Figures and Tables

**Table 1:** Nominal and real compositions of as-prepared (Co_75_Ti_25_)_100−x_B_x_ systems.

**Nominal Composition of the Starting Materials (at%)**							
**B (x)**	**2**	**5**	**10**	**15**	**20**	**25**	**30**
Co	73.50	71.25	67.50	63.75	60	56.25	52.50
Ti	24.5	23.75	22.50	21.25	20	18.75	17.50
**Real Composition, after MA Experiments (at%)**							
B (x)	2.01	5.05	10.05	15.00	19.98	24.93	30.02
Co	73.47	71.22	67.48	63.72	59.94	56.28	52.52
Ti	24.52	23.73	22.47	21.28	20.08	18.79	17.46

**Table 2:** FE-HRTEM/EDS elemental analysis of selected zones shown in Figure 6a.

Alloying Elements (at%)			
Zone	Co	Ti	B
I	56.24	18.72	25.04
II	56.19	18.82	24.99
III	56.22	18.73	25.05
IV	56.29	18.80	24.91
V	56.20	18.76	25.04
VI	56.21	18.70	25.09

**Table 3:** FE-HRTEM/EDS elemental analysis of selected spots shown in Figure 7a.

Alloying Elements (at%)			
Zone	Co	Ti	B
I	45.11	12.51	42.38
II	49.83	15.32	34.85
III	52.07	13.66	38.41
IV	52.39	18.92	28.69
V	49.28	17.95	32.77
VI	38.35	14.67	46.98
